# Seroprevalence of anti-SARS-CoV-2 antibodies and cross-variant neutralization capacity after the Omicron BA.2 wave in Geneva, Switzerland: a population-based study

**DOI:** 10.1016/j.lanepe.2022.100547

**Published:** 2022-12-01

**Authors:** María-Eugenia Zaballa, Javier Perez-Saez, Carlos de Mestral, Nick Pullen, Julien Lamour, Priscilla Turelli, Charlène Raclot, Hélène Baysson, Francesco Pennacchio, Jennifer Villers, Julien Duc, Viviane Richard, Roxane Dumont, Claire Semaani, Andrea Jutta Loizeau, Clément Graindorge, Elsa Lorthe, Jean-François Balavoine, Didier Pittet, Manuel Schibler, Nicolas Vuilleumier, François Chappuis, Omar Kherad, Andrew S. Azman, Klara M. Posfay-Barbe, Laurent Kaiser, Didier Trono, Silvia Stringhini, Idris Guessous, Specchio-COVID19 study group, Isabelle Arm-Vernez, Andrew S Azman, Delphine Bachmann, Antoine Bal, Jean-François Balavoine, Michael Balavoine, Rémy P Barbe, Hélène Baysson, Lison Beigbeder, Julie Berthelot, Patrick Bleich, Livia Boehm, Gaëlle Bryand, François Chappuis, Prune Collombet, Sophie Coudurier-Boeuf, Delphine Courvoisier, Alain Cudet, Vladimir Davidovic, Carlos de Mestral, Paola D'ippolito, Richard Dubos, Roxane Dumont, Isabella Eckerle, Nacira El Merjani, Antoine Flahault, Natalie Francioli, Marion Frangville, Clément Graindorge, Idris Guessous, Séverine Harnal, Samia Hurst, Laurent Kaiser, Omar Kherad, Julien Lamour, Pierre Lescuyer, Arnaud G L'Huillier, François L'Huissier, Andrea Jutta Loizeau, Elsa Lorthe, Chantal Martinez, Lucie Ménard, Ludovic Metral-Boffod, Alexandre Moulin, Mayssam Nehme, Natacha Noël, Francesco Pennacchio, Javier Perez-Saez, Didier Pittet, Klara M Posfay-Barbe, Géraldine Poulain, Caroline Pugin, Nick Pullen, Viviane Richard, Frederic Rinaldi, Déborah Rochat, Irine Sakvarelidze, Khadija Samir, Hugo Santa Ramirez, Etienne Satin, Philippe Schaller, Manuel Schibler, Stephanie Schrempft, Claire Semaani, Silvia Stringhini, Stéphanie Testini, Didier Trono, Déborah Urrutia-Rivas, Charlotte Verolet, Pauline Vetter, Jennifer Villers, Guillemette Violot, Nicolas Vuilleumier, Ania Wisniak, Sabine Yerly, María-Eugenia Zaballa

**Affiliations:** a Unit of Population Epidemiology, Division of Primary Care Medicine, Geneva University Hospitals, Geneva, Switzerland; b Department of Epidemiology, Johns Hopkins Bloomberg School of Public Health, Baltimore, MD, United States; c University Centre for General Medicine and Public Health, University of Lausanne, Lausanne, Switzerland; d School of Life Sciences, Ecole Polytechnique Fédérale de Lausanne, Lausanne, Switzerland; e Department of Health and Community Medicine, Faculty of Medicine, University of Geneva, Geneva, Switzerland; f Department of Medicine, Faculty of Medicine, University of Geneva, Geneva, Switzerland; g Infection Control Program and World Health Organization Collaborating Centre on Patient Safety, Geneva University Hospitals, Geneva, Switzerland; h Division of Laboratory Medicine, Department of Diagnostics, Geneva University Hospitals, Geneva, Switzerland; i Division and Department of Primary Care Medicine, Geneva University Hospitals, Geneva, Switzerland; j Division of Internal Medicine, Hôpital de la Tour, Geneva, Switzerland; k Department of Woman, Child, and Adolescent Medicine, Geneva University Hospitals, Geneva, Switzerland; l Department of Pediatrics, Gynecology & Obstetrics, Faculty of Medicine, University of Geneva, Geneva, Switzerland; m Division of Infectious Diseases, Department of Medicine, Geneva University Hospitals, Geneva, Switzerland; n Geneva Centre for Emerging Viral Diseases, Geneva University Hospitals, Geneva, Switzerland

**Keywords:** Anti-SARS-CoV-2 antibodies, Neutralizing antibodies, Variants of concern, Omicron, Seroprevalence, Switzerland

## Abstract

**Background:**

More than two years into the COVID-19 pandemic, most of the population has developed anti-SARS-CoV-2 antibodies from infection and/or vaccination. However, public health decision-making is hindered by the lack of up-to-date and precise characterization of the immune landscape in the population. Here, we estimated anti-SARS-CoV-2 antibodies seroprevalence and cross-variant neutralization capacity after Omicron became dominant in Geneva, Switzerland.

**Methods:**

We conducted a population-based serosurvey between April 29 and June 9, 2022, recruiting children and adults of all ages from age-stratified random samples of the general population of Geneva, Switzerland. We tested for anti-SARS-CoV-2 antibodies using commercial immunoassays targeting either the spike (S) or nucleocapsid (N) protein, and for antibody neutralization capacity against different SARS-CoV-2 variants using a cell-free Spike trimer-ACE2 binding-based surrogate neutralization assay. We estimated seroprevalence and neutralization capacity using a Bayesian modeling framework accounting for the demographics, vaccination, and infection statuses of the Geneva population.

**Findings:**

Among the 2521 individuals included in the analysis, the estimated total antibodies seroprevalence was 93.8% (95% CrI 93.1–94.5), including 72.4% (70.0–74.7) for infection-induced antibodies. Estimates of neutralizing antibodies in a representative subsample (N = 1160) ranged from 79.5% (77.1–81.8) against the Alpha variant to 46.7% (43.0–50.4) against the Omicron BA.4/BA.5 subvariants. Despite having high seroprevalence of infection-induced antibodies (76.7% [69.7–83.0] for ages 0–5 years, 90.5% [86.5–94.1] for ages 6–11 years), children aged <12 years had substantially lower neutralizing activity than older participants, particularly against Omicron subvariants. Overall, vaccination was associated with higher neutralizing activity against pre-Omicron variants. Vaccine booster alongside recent infection was associated with higher neutralizing activity against Omicron subvariants.

**Interpretation:**

While most of the Geneva population has developed anti-SARS-CoV-2 antibodies through vaccination and/or infection, less than half has neutralizing activity against the currently circulating Omicron BA.5 subvariant. Hybrid immunity obtained through booster vaccination and infection confers the greatest neutralization capacity, including against Omicron.

**Funding:**

General Directorate of Health in Geneva canton, Private Foundation of the Geneva University Hospitals, 10.13039/501100000780European Commission (“CoVICIS” grant), and a private foundation advised by CARIGEST SA.

**Research in context**

**Evidence before this study**

Although prevalence of anti-SARS-CoV-2 antibodies developed through infection and/or vaccination is thought to be high in most settings, the extent of population-level neutralizing capacity against past and current variants of concern (VOCs) is unknown. We searched PubMed, medRxiv, and bioRxiv using no language restrictions and search query [“COVID-19” AND “neutrali∗” AND (“prevalence” OR “seroprevalence”)] in the Title and Abstract fields on July 20, 2022. Most relevant seroprevalence studies we found focused on specific populations, particularly health care workers. Most studies reporting population-level seroprevalence estimates along with data from neutralization assays used the latter for diagnostic confirmation only. We found four studies reporting population-level seroprevalence of neutralizing antibodies, however, these were all conducted before June 2021 and assessed neutralization against the ancestral SARS-CoV-2 strain only.

**Added value of this study**

We here quantified both anti-SARS-CoV-2 antibody presence and neutralizing capacity against VOCs, including five Omicron subvariants, through a population-based serosurvey in the canton of Geneva, Switzerland. Our findings reveal that more than nine in ten (93.8%) individuals in the population have antibodies, including seven in ten (72.4%) individuals with antibodies of infection origin. However, neutralizing capacity is significantly lower ranging from 78.3% against the ancestral D614G strain to 46.7% for the currently dominating Omicron BA.5 subvariant, with large differences between age groups. Highest neutralizing capacity against Omicron subvariants was associated with vaccination booster alongside recent infection. These estimates provide an up-to-date and population-level picture of the SARS-CoV-2 immune landscape as shaped by age-specific infection and vaccination patterns.

**Implications of all the available evidence**

Our results show that through vaccination and several variant-driven pandemic waves, seroprevalence of anti-SARS-CoV-2 antibodies is high in the population, but that neutralizing capacity is variant-specific and determined by infection history and vaccination status, yielding strong differences between age groups in the general population. They also highlight the importance of booster vaccination, which appears to confer the highest neutralizing capacity, including against Omicron subvariants. These results may help prioritizing public health interventions against current and future variants as the COVID-19 pandemic progresses.

## Introduction

By the end of 2021, most of the world's population had developed antibodies against the severe acute respiratory syndrome coronavirus 2 (SARS-CoV-2), through infection, vaccination, or both.^1^, ^2^, ^3^ In high-income countries, vaccination programs have contributed to high prevalence of anti-SARS-CoV-2 antibodies particularly among elderly people and individuals with chronic conditions,^1^, ^2^, ^3^ two groups with high risk of severe COVID-19, hospitalization, and death.^4^^,^^5^ While population-based serosurveys remain important to monitor the pandemic,^6^ they fall short in shedding light on the state of population-level protection against infection from current SARS-CoV-2 variants.^7^, ^8^, ^9^, ^10^ In fact, studies on neutralizing antibodies, primarily based on small and non-representative samples,^11^ have shown that neutralizing capacity depends strongly on how antibodies are mounted (through vaccination and/or infection; and the number, duration and severity of infection episodes) and may differ between variants.^8^, ^9^, ^10^, ^11^ This has implications for current immune landscapes shaped by complex age-specific vaccination and infection patterns. Vaccination-induced antibodies have demonstrated high neutralizing capacity against previously predominant variants (ancestral D614G, Alpha, and Delta), but substantially less neutralizing capacity against more recent and currently dominant Omicron subvariants^8^^,^^9^^,^^12^^,^^13^—even though, importantly, protection against severe disease, hospitalization, and death remains high.^14^^,^^15^ Simultaneously, antibodies developed through infection by the Omicron BA.1 subvariant have shown reduced neutralizing capacity against most other variants of concern (VOCs).^8^^,^^9^^,^^13^^,^^16^^,^^17^

From a public health standpoint, having up-to-date estimates of antibody seroprevalence, their origin, and their neutralizing capacity against diverse circulating VOCs is critically important to disseminate public health messages to the population and to inform and adapt decisions on vaccination strategies, mask requirements, and other preventive measures. Such evidence-based decisions are needed to minimize the number of people falling ill with severe disease, requiring hospitalization, and dying during the current wave, driven by the highly contagious Omicron BA.5 subvariant, as well as future pandemic waves.

To our knowledge, at the time of writing, there are no population-based seroprevalence estimates of neutralizing antibodies against main VOCs, particularly after the Omicron variant became predominant. To fill this gap, we recruited a representative sample of the general population of Geneva, Switzerland, and assessed seroprevalence of anti-SARS-CoV-2 antibodies and their neutralizing capacity against SARS-CoV-2 VOCs 28 months after the first confirmed case in the country and 5 months after the Omicron BA.1 subvariant became dominant.^18^

With a population of about 500,000, the canton of Geneva, Switzerland, has had 261,946 confirmed cases (448 per 1000 inhabitants) and 883 deaths reported by July 21st, 2022.^19^ Our previous serosurvey revealed that by June–July 2021, around two thirds of the population had developed anti-SARS-CoV-2 antibodies following vaccination and/or infection, half of which having antibodies of infection origin.^20^ Since then, eligibility for vaccination against COVID-19 has progressively expanded in Geneva to cover most age groups, including ages 12–15 years since June 2021 and ages 5–11 years since January 2022 (appendix p 6).

## Methods

### Study design

Between April 29 and June 9, 2022, we recruited participants from a random sample of individuals aged ≥6 months provided by the Geneva cantonal population registry (*Office cantonal de la population et des migrations*), and from an age- and sex-stratified random sample of adults who participated in at least one of our previous serosurveys (appendix p 7).^20^, ^21^, ^22^ Newly selected individuals were invited by letter, while returning participants were invited by letter or email when available. A written reminder was sent to all non-responding individuals and a phone call was made to all whose phone number was available. Children and teenagers (<18 years) were invited to participate with members of their household. Participation rates differed across age groups and depending on previous participation: 12.5% for those aged <18 years, 19.5% for those aged 18–64 years and 37.4% for those aged ≥65 years among newly invited participants; and 59.4% for those aged 18–64 years and 84.2% for those aged ≥65 years among returning participants (in all cases, after excluding not only ineligible individuals but also those hospitalized or in bad health conditions or not in Geneva during the whole study) (appendix pp 7–8). Participants provided a venous blood sample and completed an online or paper questionnaire that collected sociodemographic and vaccination information and COVID-19-related medical history. Informed written consent was obtained from all participants. The Geneva Cantonal Commission for Research Ethics approved this study (Project N° 2020-00881).

For seroprevalence analyses, anti-SARS-CoV-2 antibodies presence was assessed in all participants while neutralizing activity against SARS-CoV-2 variants was only tested in a subset of them, the newly selected participants. This newly randomly selected sample included individuals of all ages, while the randomly selected sample of returning participants excluded those aged <18 years (appendix p 7).

### Immunoassays

To detect anti-SARS-CoV-2 antibodies, we used two commercially available immunoassays: the Roche Elecsys anti-SARS-CoV-2 S and anti-SARS-CoV-2 N immunoassays (Roche Diagnostics, Rotkreuz, Switzerland), which detect immunoglobulins (IgG/A/M) against the receptor binding domain of the virus spike (S) protein (#09 289 275 190, Roche-S) and the virus nucleocapsid (N) protein (#09 203 079 190, Roche-N), respectively. Both assays have high accuracy and have been validated in multiple settings, including our previous serosurveys.^20^, ^21^, ^22^ We defined seropositivity using the manufacturer's provided cut-off values of titer ≥0.8 U/mL for the Roche-S, and cut-off index ≥1.0 for the Roche-N immunoassays.

### S^3^-cell free neutralization assay

To assess anti-SARS-CoV-2 antibody neutralizing activity, we used the S^3^-ACE2 neutralization assay.^23^^,^^24^ Production and purification of trimeric Spike variants (D614G [B.1], Alpha [B1.1.7], Beta [B.1.351], Gamma [P.1], Delta [B.1.617.2], Iota [B.1.526], Kappa [B.1.617.1], Lambda [C.37], and Omicron [BA.1, BA.2, BA.2.12.1 and BA.4/BA.5]) and ACE2 mouse Fc fusion protein as well as Spike protein-beads coupling were performed as previously described in a validation study.^23^ Variant-specific validation tests against cell-based neutralization experiments are performed as lab routine on a small subset of serum samples if the corresponding live replicating virus lineage/sublineage is available, *i.e.* for four of the variants here analyzed (D614G, Alpha, Delta and Omicron BA.1). Neutralization assays were done in 96-well plates, where 10 multiplexed Spike variants were incubated with sera, as described.^23^ Briefly, a volume of 5 μL of serum per well was used for the starting dilution and a total of 6 serial dilutions (1:10, 1:30, 1:90, 1:270, 1:810, 1:7290) of sera were incubated 1 h with the Spike proteins before ACE2 mouse Fc fusion protein was added, and binding detected with an anti-mouse IgG-PE secondary antibody (eBioscience, Thermo Fisher Scientific, catalogue #12-4010-87). Control wells were included on each 96-well plate with each variant Spike-coupled beads alone. Plates were read with a Luminex 200 instrument and mean fluorescence intensity (MFI) for beads without serum was averaged and used as the 100% binding signal for the ACE2 receptor to the bead-coupled Spike trimer. MFI obtained for D614G Spike using a high concentration of imdevimab (RGN10987, 1 μg/mL), a monoclonal antibody known to neutralize the ancestral strain, was used as the maximum inhibition signal.^25^ The percent blocking of the Spike trimer-ACE2 interaction was calculated using the formula: % Inhibition = (1- ([MFI Test dilution – MFI Max inhibition]/[MFI Max binding – MFI Max inhibition]) × 100). Serum dilution response inhibition curves were generated using GraphPad Prism 8.3.0. NonLinear four-parameter curve fitting analysis of the agonist versus response, and ED50% values (mean serum dilution needed to achieve 50% neutralization) were extracted using an in-house script (https://doi.org/10.5281/zenodo.7124818). Neutralizing capacity was assessed against the ancestral variant (D614G), the Alpha, Beta, Gamma, Delta, Iota, Kappa, and Lambda variants, and the Omicron BA.1, BA.2, BA.2.12.1, and BA.4/BA.5 subvariants (Spike proteins of BA.4 and BA.5 subvariants share identical sequences^17^ and thus results of the cell-free surrogate neutralization assay apply to both).

### Statistical analyses

To estimate seroprevalence of anti-SARS-CoV-2 antibodies (% and 95% credible intervals [95% CrI]), we used a Bayesian modeling framework jointly inferring anti-N and anti-S presence while accounting for age, sex, immunoassay performance, and household clustering following our previous work.^20^ Since the vaccines used to date in Geneva do not elicit a response to the SARS-CoV-2 N protein,^26^ we used participants' two-marker antibody profiles to estimate the proportion of those having anti-SARS-CoV-2 antibodies from any origin (vaccination and/or infection) and those having antibodies due to infection (who could be vaccinated or not). To obtain population-level estimates of total and infection-induced antibodies seroprevalence, we post-stratified model estimates to account for the sex and age distribution of the Geneva general population and for household clustering of infection and vaccination. Separately, in a model including only participants aged ≥18 years, we additionally post-stratified for educational level. We further developed a Bayesian logistic model to estimate the seroprevalence of neutralizing antibodies (% and 95% CrI), accounting for age, sex, and infection (uninfected, latest infected by a pre-Omicron variant, latest infected by an Omicron subvariant) and vaccination status (unvaccinated, vaccinated without booster, vaccinated with booster). The term ‘booster’ refers to a vaccine dose received several months after completion of primary vaccination: it could mean having received a third vaccine dose after two-dose primary vaccination or having received a second vaccine dose after one-dose primary vaccination post-infection. Data on infection dates and vaccination was self-reported by the participants at the moment of the blood drawing (appendix pp 3–5). Since Omicron became the dominant circulating variant in the Geneva region by late December 2021,^18^ we assumed infections were due to Omicron (no subvariants distinction) if the participant reported having had a COVID-19 diagnostic positive test (PCR or rapid antigen test, including self-tests) after January 1st, 2022. Our base model assumed additive contributions of vaccination and infection and was fit to each variant separately. As sensitivity analysis, we fit three additional sets of models: the first with interaction terms between infection and vaccination status, the second with overdispersion in neutralizing capacity, and the third with differential surrogate neutralization test performance for children under the age of 12 years versus older individuals. Model selection was performed based on estimated leave-one-out cross-validation error.^27^ To obtain population-level estimates of neutralizing antibodies seroprevalence, we post-stratified model estimates to account for the age and sex distribution and vaccination and infection statuses of the general population of Geneva. Full details of the statistical models are provided in the supplement (appendix pp 3–5).

### Role of the funding source

The funding sources had no role in the study design, methodology, data collection or analysis, results interpretation, manuscript writing or decision to submit manuscript for publication.

## Results

### Overall anti-SARS-CoV-2 seroprevalence estimates

Our analytical sample for seroprevalence estimation comprised 2521 participants (appendix p 7), of whom 55.2% were women, 21.4% were aged <18 years and 14.2% were aged ≥65 years (appendix p 9). Among adults, 11.3% had a primary education level and 56.9% had a tertiary education level, compared with 26.6% and 41.5%, respectively, in the general population of Geneva (appendix p 19). Overall, 75.4% of participants declared having received at least one COVID-19 vaccine dose at the time of their recruitment in the study, compared with 71.1% in the Geneva general population (appendix p 20); 96.9% of all participants tested positive for anti-S antibodies, and 70.0% tested positive for anti-N antibodies (Table 1; appendix p 10).

**Table 1:** Demographic characteristics of sample, serological results, and seroprevalence estimates in Geneva, Switzerland, April 29 to June 9, 2022.

	Participants	Vaccinated (self-reported)^a^	Seropositive^b^		Seroprevalence^c^	
			Anti-SARS-CoV-2 S protein	Anti-SARS-CoV-2 N protein	Antibodies of any origin % (95% CrI)	Antibodies of infection origin % (95% CrI)
**Total**	2521	1902 (75.4)	2442 (96.9)	1765 (70.0)	93.8 (93.1–94.5)	72.4 (70.0–74.7)
**Men**	1129 (44.8)	844 (74.8)	1090 (96.5)	775 (68.6)	93.3 (92.4–94.2)	71.7 (68.5–74.9)
**Women**	1392 (55.2)	1058 (76.0)	1352 (97.1)	990 (71.1)	94.2 (93.4–95.0)	73.1 (70.2–75.8)
**Age**, y						
0-5	144 (5.7)	3 (2.1)	112 (77.8)	107 (74.3)	77.2 (70.3–83.3)	76.7 (69.7–83.0)
6-11	242 (9.6)	23 (9.5)	231 (95.5)	217 (89.7)	91.0 (87.1–94.4)	90.5 (86.5–94.1)
12-17	155 (6.1)	95 (61.3)	153 (98.7)	126 (81.3)	93.4 (90.3–96.1)	86.4 (80.2–91.9)
18-24	169 (6.7)	150 (88.8)	169 (100.0)	135 (79.9)	95.0 (93.0–96.9)	84.0 (77.8–90.1)
25-34	267 (10.6)	239 (89.5)	263 (98.5)	209 (78.3)	94.9 (93.7–95.9)	78.9 (74.3–83.3)
35-49	787 (31.2)	706 (89.7)	780 (99.1)	554 (70.4)	95.0 (94.2–95.7)	74.4 (70.5–78.1)
50-64	400 (15.9)	349 (87.2)	387 (96.8)	254 (63.5)	95.4 (94.7–96.1)	67.7 (62.5–72.6)
65-74	183 (7.3)	173 (94.5)	179 (97.8)	92 (50.3)	95.1 (94.3–95.9)	55.0 (47.3–62.5)
≥75	174 (6.9)	164 (94.3)	168 (96.6)	71 (40.8)	96.7 (96.2–97.1)	45.9 (38.3–53.7)

Data are n (%) unless otherwise stated. CrI: credible interval; N: nucleocapsid protein; S: spike protein; SARS-CoV-2: severe acute respiratory syndrome coronavirus 2.

a Self-reported having received at least 1 dose of the COVID-19 vaccine before blood sample was drawn.

b Serology based on Roche Elecsys anti-SARS-CoV-2 S immunoassay and N immunoassay, respectively.

c Seroprevalence estimates reported as % and 95% credible interval, adjusted for test performance of both immunoassays and post-stratified to account for the sex and age distribution of the Geneva general population and for household clustering of infection and vaccination. Seroprevalence of any antibodies is based on proportion of participants with any anti-SARS-CoV-2 antibodies, additionally post-stratified to the vaccination data in the general population of Geneva; seroprevalence of infection antibodies is based on proportion of participants who were naturally infected (but could also have been vaccinated).

**Fig. 1:**
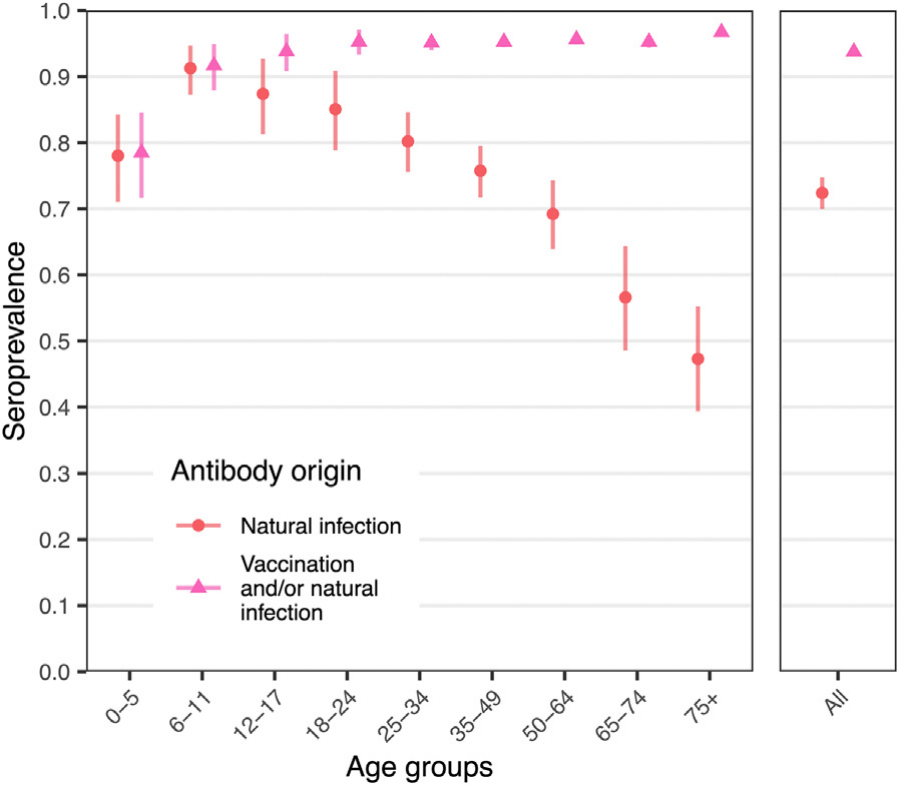
**Seroprevalence of anti-SARS-CoV-2 antibodies in the general population of Geneva, Switzerland, April 29 to June 9, 2022.** Seroprevalence estimates in total sample and by age group (in years) and origin of antibody response. Symbols indicate the antibody origin: *dot* indicates antibodies developed after infection; *triangle* indicates antibodies developed after infection and/or vaccination. Vertical bars represent 95% credible intervals.

After accounting for the Geneva population demographics and vaccination status, the overall seroprevalence of anti-SARS-CoV-2 antibodies developed through vaccination and/or infection was 93.8% (95% CrI: 93.1–94.5), with no difference between men and women (Table 1). Estimates varied only slightly across age groups except the youngest; overall seroprevalence among children aged 0–5 years was 77.2% (70.3–83.3) while it was 91.0% (87.1–94.4) among children aged 6–11 years, progressively increasing with age to reach 96.7% (96.2–97.1) among adults aged ≥75 years. The overall seroprevalence of infection-induced antibodies was 72.4% (70.0–74.7), also being similar between men and women, but varying considerably across age groups. Among children aged 0–5 years, the estimate was 76.7% (69.7–83.0), while it was highest among children aged 6–11 years at 90.5% (86.5–94.1), markedly decreasing with age and being lowest at 45.9% (38.3–53.7) among adults aged ≥75 years (Fig. 1; appendix pp 11, 21). We found no meaningful differences in vaccination rate or seroprevalence estimates according to educational level (appendix pp 21–22).

### Seroprevalence estimates and determinants of anti-SARS-CoV-2 antibodies neutralizing capacity

Our analytical sample for assessing anti-SARS-CoV-2 neutralization activity included a subset of 1160 participants (appendix p 7), of whom 54.4% were women, 28.2% aged <18 years and 19.3% aged ≥65 years (appendix pp 9, 24). Overall anti-SARS-CoV-2 seroprevalence estimates on this subsample were similar to those obtained on the main study sample (appendix p 24). Distribution of ED50% values obtained on this subsample for all tested SARS-CoV-2 variants using our cell-free surrogate neutralization assay is shown in Fig. S7 (appendix p 12).

**Fig. 2:**
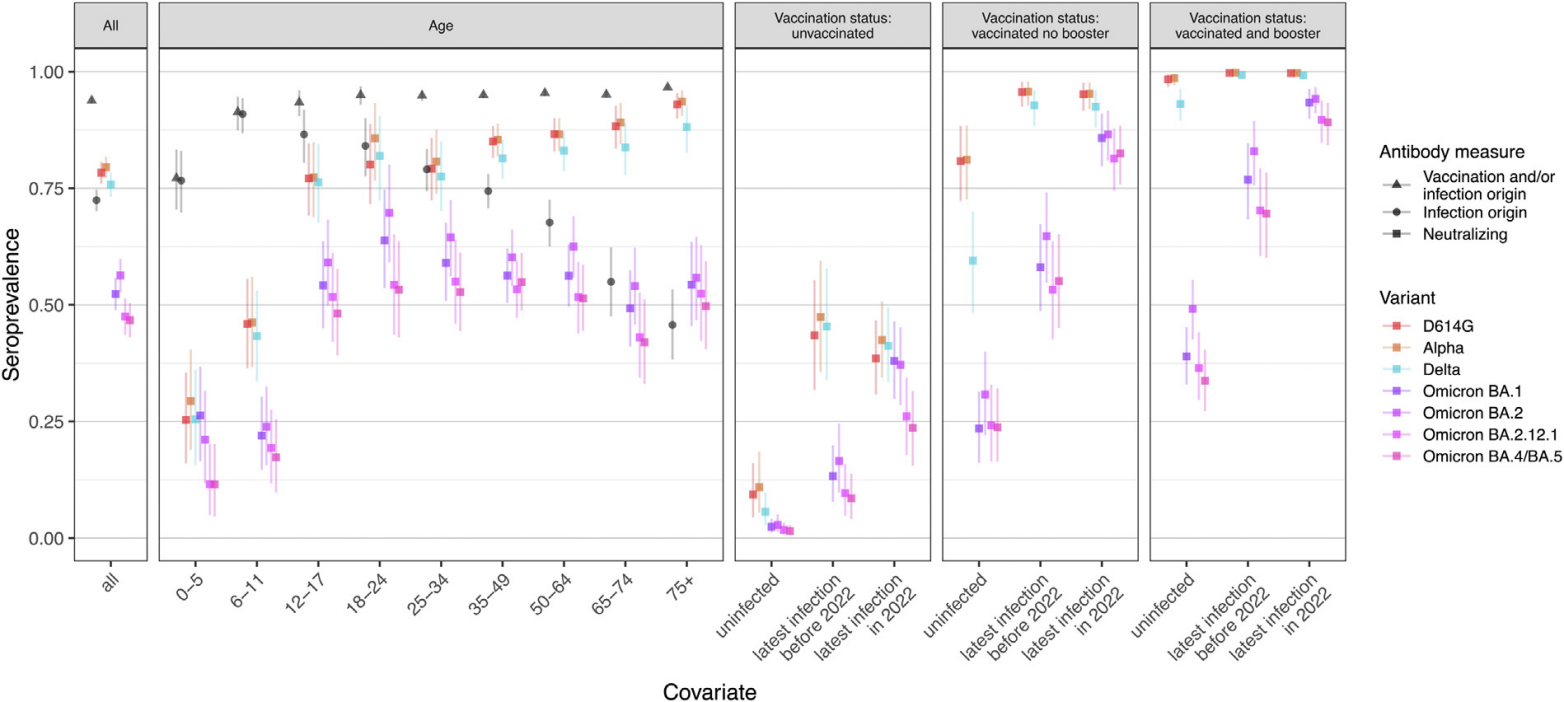
**Seroprevalence of neutralizing antibodies against main SARS-CoV-2 variants in the general population of Geneva, Switzerland, April 29 to June 9, 2022.** Panels show the effect of covariates tested in the model: age and infection and vaccination statuses (self-reported)—though sex was included as covariate, it showed no apparent effect, so it is excluded here, but estimates are shown in appendix pp 13, 25–27. Estimates of neutralizing capacity against Beta, Gamma, and Lambda variants are shown in appendix pp 13, 25–27. Global seroprevalence estimates for anti-S and anti-N antibodies are included in black in the two left panels for comparison purposes. Symbols indicate the antibody origin: *dot* indicates antibodies developed after infection (anti-N); *triangle* indicates antibodies developed after infection and/or vaccination (anti-S); and *square* indicates neutralizing antibodies (cell-free surrogate neutralization assay). Vertical bars represent 95% credible intervals.

After accounting for the general population demographic and vaccination and infection status distributions, population-level neutralizing capacity was variant-specific (Fig. 2; appendix p 13): while 75–80% of the population had neutralizing antibodies against the ancestral D614G, Alpha, and Delta variants, seroprevalence of neutralizing antibodies against all tested Omicron subvariants was lower than 60% (Fig. 2; appendix pp 13, 25-27).

Among children aged 0–5 years, estimated seroprevalence of neutralizing antibodies was 29.3% (18.9–40.4) and 26.3% (16.4–36.7) against Alpha and Omicron BA.1, respectively, but 11.5% (4.7–20.2) against Omicron BA.4/BA.5. For children aged 6–11 years, seroprevalence of neutralizing antibodies ranged from 46.2% (36.7–56.0) against Alpha to 17.3% (9.8–25.5) against Omicron BA.4/BA.5. Starting with age 12 years, seroprevalence of neutralizing antibodies against D614G, Alpha and Delta was markedly higher: around 75% among adolescents aged 12–17 years, and around 90% among individuals aged ≥75 years. However, seroprevalence of neutralizing antibodies against the Omicron subvariants remained relatively similar, and lower, across these age groups, being 48.2% (39.1–57.7) and 49.7% (40.5–59.3) against BA.4/BA.5 among individuals aged 12–17 years and ≥75 years, respectively (Fig. 2; appendix pp 13, 25–27).

Seroprevalence of neutralizing antibodies varied considerably according to vaccination and infection statuses (Fig. 2; appendix pp 13, 25–27). In general, neutralizing capacity against D614G, Alpha, and Delta variants was substantial (>90%) among vaccinated individuals having received booster vaccination, regardless of infection status. However, among vaccinated individuals without booster vaccination, neutralizing capacity was decreased (reaching 60%) if uninfected. Consistently, in multivariable analyses, having received booster vaccination showed the strongest association with neutralizing capacity; for instance, boosted individuals had 14.2 (95% Cr: 6.4–28.0) times greater odds of having neutralizing antibodies against Delta, and 2.2 (1.4–3.4) times the odds of having antibodies with neutralizing capacity against Omicron BA.4/BA.5, compared with vaccinated individuals without booster vaccination (Table 2). Regarding infection, compared with individuals last infected before 2022, those infected in 2022 had more than three times greater odds of having neutralizing antibodies against the Omicron subvariants. Finally, regardless of variant, unvaccinated individuals had substantially lower odds of having neutralizing antibodies (Table 2).

**Table 2:** Association between individuals’ attributes and neutralizing capacity against main SARS-CoV-2 variants in the general population of Geneva, Switzerland, April 29 to June 9, 2022.

	SARS-CoV-2 variant						
	D614G	Alpha	Delta	Omicron BA.1	Omicron BA.2	Omicron BA.2.12.1	Omicron BA.4/BA.5
**Men**	0.83 (0.51–1.26)	0.84 (0.52–1.26)	0.90 (0.59–1.34)	0.97 (0.69–1.31)	0.92 (0.65–1.27)	0.98 (0.67–1.39)	1.07 (0.75–1.49)
**Women**	1 (ref)	1 (ref)	1 (ref)	1 (ref)	1 (ref)	1 (ref)	1 (ref)
**Age group**, y							
0-5	0.66 (0.28–1.31)	0.74 (0.31–1.46)	0.69 (0.30–1.36)	1.39 (0.59–2.74)	0.99 (0.38–2.05)	0.68 (0.24–1.51)	0.68 (0.24–1.52)
6-11	1.34 (0.66–2.43)	1.22 (0.60–2.20)	1.22 (0.60–2.22)	0.89 (0.42–1.67)	0.98 (0.45–1.82)	1.09 (0.48–2.10)	0.95 (0.41–1.86)
12-17	2.20 (0.98–4.25)	2.05 (0.91–3.98)	2.50 (1.17–4.77)	2.41 (1.22–4.27)	2.75 (1.39–4.89)	2.63 (1.33–4.72)	2.03 (1.01–3.75)
18-24	1.38 (0.49–3.18)	2.40 (0.79–6.05)	2.10 (0.76–4.75)	2.41 (1.11–4.65)	2.91 (1.32–5.68)	1.64 (0.80–3.02)	1.41 (0.67–2.64)
25-34	0.86 (0.32–1.97)	1.00 (0.37–2.27)	1.09 (0.44–2.37)	1.34 (0.70–2.33)	1.56 (0.83–2.72)	1.28 (0.66–2.27)	1.00 (0.52–1.78)
35-49	1 (ref)	1 (ref)	1 (ref)	1 (ref)	1 (ref)	1 (ref)	1 (ref)
50-64	0.75 (0.33–1.55)	0.72 (0.32–1.44)	0.80 (0.40–1.47)	0.95 (0.55–1.54)	1.03 (0.61–1.65)	0.85 (0.48–1.36)	0.77 (0.44–1.26)
65-74	0.74 (0.22–2.00)	0.85 (0.27–2.25)	0.73 (0.29–1.62)	0.77 (0.41–1.29)	0.72 (0.38–1.24)	0.61 (0.32–1.06)	0.54 (0.27–0.98)
≥75	0.96 (0.28–2.59)	1.19 (0.33–3.24)	0.78 (0.29–1.70)	0.99 (0.52–1.72)	0.74 (0.40–1.30)	1.00 (0.51–1.82)	0.83 (0.43–1.51)
**Infection status**^a^							
Uninfected	0.18 (0.08–0.35)	0.18 (0.08–0.34)	0.10 (0.05–0.18)	0.20 (0.12–0.30)	0.21 (0.12–0.33)	0.25 (0.14–0.38)	0.23 (0.13–0.35)
Pre-2022 infection	1 (ref)	1 (ref)	1 (ref)	1 (ref)	1 (ref)	1 (ref)	1 (ref)
2022 infection	0.89 (0.49–1.56)	0.91 (0.49–1.55)	0.95 (0.52–1.57)	4.62 (2.77–7.39)	3.67 (2.15–6.02)	3.99 (2.35–6.65)	3.91 (2.36–6.43)
**Vaccination status**^b^							
Unvaccinated	0.03 (0.01–0.05)	0.03 (0.02–0.06)	0.05 (0.03–0.09)	0.10 (0.05–0.16)	0.09 (0.05–0.15)	0.08 (0.04–0.14)	0.07 (0.03–0.12)
Vaccinated, no booster	1 (ref)	1 (ref)	1 (ref)	1 (ref)	1 (ref)	1 (ref)	1 (ref)
Vaccinated, booster	21.99 (7.90–53.43)	25.21 (8.23–62.8)	14.19 (6.44–28.0)	2.89 (1.78–4.56)	3.35 (2.05-5.30)	2.51 (1.56–3.96)	2.20 (1.36–3.39)

Data are odds ratio (95% credible interval) of having neutralizing antibodies against a SARS-CoV-2 variant. Only main variants having been detected at significant proportions in the canton of Geneva are included in this table. SARS-CoV-2: severe acute respiratory syndrome coronavirus 2.

a Self-reported most recent infection via a COVID-19 diagnostic positive test (PCR or rapid antigen test, including self-tests).

b Vaccination status based on self-reported number of doses received and dates. The term 'booster' refers to a vaccine dose received several months after completion of primary vaccination: it could mean having received a third vaccine dose after two-dose primary vaccination or having received a second vaccine dose after one-dose primary vaccination post-infection.

## Discussion

This serosurvey found that by April–June 2022, 93.8% of the Geneva population had developed antibodies against SARS-CoV-2 after vaccination and/or infection. Yet the proportion of the population with neutralizing antibodies varied considerably across SARS-CoV-2 variants, from more than three quarters against Alpha to less than half against the currently circulating Omicron BA.5 subvariant, with particularly lower proportions in children <12 years and unvaccinated individuals who were last infected before Omicron became dominant.

The seroprevalence of total antibodies estimated in this study is slightly lower than the 97.5–98.8% found in two other Swiss sample populations by the end of March 2022,^28^ and the 94.7–96.1% reported in the United Kingdom by mid-July 2022,^29^ but these estimations only included individuals aged ≥16 years. We also found that 72.4% of the population had been infected—a 42.5 percentage point increase from the 29.9% seroprevalence reported by June–July, 2021^20^ (appendix p 28). This important increase in seroprevalence of infection-induced antibodies within a 11-month period was largest among children aged 0–5 years (55.8 percentage point increase) and 6–11 years (59.5 percentage point increase), indicating that the Delta-dominant and notably the Omicron-dominant pandemic waves in Geneva particularly affected children,^30^ as observed in other countries.^29^^,^^31^ Conversely, this increase in infection-induced antibodies does not appear to have translated to a corresponding level of neutralizing capacity; for instance, while three quarters of children aged 0–5 years had developed antibodies through infection, only around one in four had neutralizing antibodies against the Delta or Omicron BA.1 or BA.2 variants. This proportion was only slightly higher among the 6–11 years old even though nine in ten children in this age group had infection-induced antibodies. These findings indicate that most children, not having received the COVID-19 vaccine and first becoming infected by Delta or Omicron BA.1 or BA.2, did not develop neutralizing capacity against earlier variants and only limited neutralizing capacity against the currently circulating BA.5 subvariant.

Our model suggests that this discrepancy can be explained by the significantly lower vaccination rates in these age groups alone, without requiring age-specific effects (95% CrI of odds ratios for all variants and for all age groups cover 1, except for the 12–17 years age group, which had higher odds of neutralization). We however note that most infected-only participants in our sample were children under the age of 12 years, which therefore limits the power to identify age-specific effects within the analysis. Although antibodies in children seem to have a lower neutralization efficacy, their immune response has been observed to resemble that of adults in cases of mild COVID-19, but to differ in moderate to severe COVID-19 and multisystem inflammatory syndrome,^32^ including a robust mucosal response to the SARS-CoV-2 virus with high levels of interferon and a different T-cell response.^32^^,^^33^ While data on these other immunity components were not available in our study, children-specific immune response may explain the consistently lower levels of severe COVID-19, hospitalizations, and death observed among children, despite much higher levels of infections during the Delta- and Omicron-driven waves.^20^

We found little age differences in seroprevalence of total antibodies among adults, in contrast to what we observed in our previous serosurvey,^20^ likely reflecting the fact that for adults vaccination has since been widely available. Simultaneously, seroprevalence of infection-induced antibodies differed markedly among age groups, peaking for the 6–11 years age group and reducing gradually with increasing age, reflecting the pattern of age-related infection risk observed across pandemic waves, preventive measures and behavior, as well as the earlier vaccination availability and higher uptake in older people.^1^^,^^3^^,^^20^^,^^31^

The finding that neutralizing capacity against the D614G, Alpha, and Delta variants was reduced among individuals infected in 2022 compared with those last infected before 2022 is in line with reports of reduced overall neutralizing capacity against pre-Omicron variants after infection by Omicron.^8^^,^^13^^,^^16^ Notably, we also found that having received booster vaccination was associated with increased neutralizing capacity against all variants, including Omicron subvariants, in agreement with previous reports.^8^^,^^9^^,^^13^^,^^16^^,^^17^ Finally, we found that being vaccinated (with or without booster) and infected in 2022, a period when Omicron was almost exclusively circulating in Geneva, was associated with increased neutralizing capacity against all Omicron subvariants, in line with previous reports.^9^^,^^13^^,^^16^^,^^17^^,^^34^ The aforementioned Swiss serosurvey reported considerably higher neutralizing capacity than what we found, but we are unable to compare findings given a lack of methodological details in the study.^28^ Potential explanations for the observed disparities include differences in the sample age composition, proportion of boosted participants and of recent Omicron-infections, as well as the threshold used to define neutralizing activity. Despite these differences, we deem our results to reliably reflect the state of neutralization capacity in the Geneva population thanks to the use of a random sample of the population together with post-stratification based on the state's demographics and population-level infection and vaccination statuses. In general, our results revealed that a combination of vaccination (with booster) and recent infection appeared to confer the highest level of neutralizing antibodies against each variant, including Omicron subvariants.

### Implications for public health and clinical practice

Our findings show that, while most of the population have developed anti-SARS-CoV-2 antibodies through infection and/or vaccination, less than half have neutralizing antibodies against the highly contagious, currently circulating Omicron BA.5 variant, including only one in four children aged <12 years. A similar pattern of total antibodies and variant-specific neutralizing antibodies in the population is likely to be present in other settings that have experienced the same successive variant-driven pandemic waves as Geneva, Switzerland. The highest level of neutralizing capacity against each variant was observed among vaccinated individuals who had received a booster dose, indicating that vaccine-induced antibodies confer substantial neutralizing capacity against pre-Omicron variants, while also maximizing neutralizing capacity against Omicron subvariants. At the same time, not surprisingly, we observed a reduced neutralizing capacity against Omicron subvariants relative to pre-Omicron variants. This suggests that updated vaccines specifically targeting the Omicron lineage may be beneficial in containing the spread of infections and their consequent health and socio-economic burden.^1^

Our findings also revealed that, while less than half of the population show neutralizing capacity against the currently dominant Omicron subvariants, a substantial proportion have neutralizing capacity against less common variants, including Beta, Gamma, and Lambda (appendix pp 12-13, 25-27). Since future VOCs may develop from or share structural characteristics with less frequent variants, monitoring the level of neutralizing capacity against them in the population may help in building scenarios for future pandemic waves.

### Strengths and limitations

This study benefits from several strengths, including the large representative sample, the recent recruitment time-frame post-Omicron BA.1 and BA.2-driven pandemic waves, the measurement of antibodies against both the SARS-CoV-2 S and N proteins as well as neutralizing antibodies against ten SARS-CoV-2 variants/subvariants, and a robust modeling framework. We also acknowledge several limitations. First, like most serosurveys,^35^ the sample only included formal residents of the canton and had a higher proportion of individuals with tertiary education than in the general population (appendix p 19). While we used education as the only socioeconomic marker, the lack of socioeconomic inequalities in our findings is consistent with previous studies in the population of Geneva and that of neighboring countries in which other socioeconomic markers were examined.^36^, ^37^, ^38^, ^39^, ^40^ We chose against using multiple imputation for the 5% of participants (n = 99) with missing education data–while this may introduce a slight bias in the education-stratified analysis, it is unlikely to have affected the conclusions drawn from it. Second, data on infection dates was self-reported by the participants at the moment of the blood drawing and only the latest known infection was included in the analysis. Third, the cut-off value for neutralizing activity was defined using pre-pandemic sera from adult donors only—no children samples were included in the validation study, as previously reported.^23^ While the level of neutralizing antibodies has been shown to be a strong marker of immune protection, it does not fully describe it, especially among children whose immune response differs from that of adults.^32^^,^^33^ Fourth, our seroprevalence estimates assume constant test sensitivity despite potential changes in test performance with time since infection. Previous studies have shown that the performance of both Roche immunoassays used in this study remains high and with a very limited decaying trend several months after infection.^41^ We therefore expect the impact of these changes to be accounted for within our Bayesian framework, which explicitly models test performance uncertainty allowing for departure from manufacturer-provided values. Lastly, due to lack of data, we did not include in our modeling framework indicators that have been shown to influence neutralizing capacity, including severity and duration of symptoms, number of infections, and interval between last infection/vaccination and blood sampling.^9^, ^10^, ^11^^,^^42^^,^^43^

### Conclusions

This study provides up-to-date seroprevalence estimates of anti-SARS-CoV-2 antibodies in a representative sample of the general population 5 months after Omicron became the dominant circulating SARS-CoV-2 variant in Geneva, Switzerland. It shows that while most of the population (notably ≥12 years of age) have neutralizing antibodies against pre-Omicron variants, the seroprevalence of antibodies with neutralizing capacity against the currently circulating and highly contagious Omicron BA.5 subvariant is low. Our findings suggest that the mass vaccination of older individuals, as well as other preventive measures and behaviors, may have protected them from infection during the Delta- and Omicron BA.1- and BA.2-driven waves. They also show that the highest level of neutralizing capacity against most VOCs is attained through hybrid immunity combining vaccination, notably including a booster dose, and recent infection. As new variants emerge driving new pandemic waves, having up-to-date snapshots of the immune landscape of the population can help develop rational risk mitigation strategies.

## Contributors

IG, SS, LK, DT, MEZ, JPS and NP designed the study. MEZ, JL, PT, CR, HB, FP, JV, JD, VR, RD, CS, AJL, CG, EL, MS, NV, OK, KMPB, LK, DT, SS and IG contributed to participants’ recruitment and/or data acquisition. NP and JPS conducted statistical analyses. CdM conducted literature review and wrote the first draft of the manuscript. All authors contributed to the interpretation of results and read and approved the final manuscript. IG, SS, MEZ, JPS, NP and JL had full access to all data in the study, and the corresponding author had final responsibility for decision to submit for publication.

## Data sharing statement

Our data are accessible to researchers upon reasonable request for data sharing to the corresponding author. All code to reproduce the analysis as well as code for the creation of a synthetic dataset on which to run the analysis code is available at: https://github.com/UEP-HUG/sp4_public. The script to process neutralizing antibody data from the Luminex instrument is available at: https://zenodo.org/record/7124818.

## Specchio-COVID19 study group

Isabelle Arm-Vernez, Andrew S Azman, Delphine Bachmann, Antoine Bal, Jean-François Balavoine, Michael Balavoine, Rémy P Barbe, Hélène Baysson, Lison Beigbeder, Julie Berthelot, Patrick Bleich, Livia Boehm, Gaëlle Bryand, François Chappuis, Prune Collombet, Sophie Coudurier-Boeuf, Delphine Courvoisier, Alain Cudet, Vladimir Davidovic, Carlos de Mestral, Paola D'ippolito, Richard Dubos, Roxane Dumont, Isabella Eckerle, Nacira El Merjani, Antoine Flahault, Natalie Francioli, Marion Frangville, Clément Graindorge, Idris Guessous, Séverine Harnal, Samia Hurst, Laurent Kaiser, Omar Kherad, Julien Lamour, Pierre Lescuyer, Arnaud G L'Huillier, François L'Huissier, Andrea Jutta Loizeau, Elsa Lorthe, Chantal Martinez, Lucie Ménard, Ludovic Metral-Boffod, Alexandre Moulin, Mayssam Nehme, Natacha Noël, Francesco Pennacchio, Javier Perez-Saez, Didier Pittet, Klara M Posfay-Barbe, Géraldine Poulain, Caroline Pugin, Nick Pullen, Viviane Richard, Frederic Rinaldi, Déborah Rochat, Irine Sakvarelidze, Khadija Samir, Hugo Santa Ramirez, Etienne Satin, Philippe Schaller, Manuel Schibler, Stephanie Schrempft, Claire Semaani, Silvia Stringhini, Stéphanie Testini, Didier Trono, Déborah Urrutia-Rivas, Charlotte Verolet, Pauline Vetter, Jennifer Villers, Guillemette Violot, Nicolas Vuilleumier, Ania Wisniak, Sabine Yerly, and María-Eugenia Zaballa.

## Declaration of interests

DT is a founder and co-chair of the Scientific Advisory Board of Aerium Therapeutics, holds stock in that company, and has two patents pending for monoclonal antibodies against SARS-CoV-2. KMPB is a member of the Advisory Boards for pneumococcal vaccine and varicella vaccine at MSD. All other authors declare that they have no competing interests.
