# Supplementary material for: Seroprevalence of anti-SARS-CoV-2 antibodies and cross-variant neutralization capacity after the Omicron BA.2 wave in Geneva, Switzerland: a population-based study
Source: Lancet Reg Health Eur. 2022 Dec 1;24:100547. doi: 10.1016/j.lanepe.2022.100547 (PMC9714630; doi:10.1016/j.lanepe.2022.100547)
Supplement: Supplementary File S2 [file mmc3.docx]

**Supplementary material caption**

**Supplementary information S1**. Study sample selection and questions given to participants that were used in the present study

**Supplementary information S2**. Overview of Statistical Frameworks

**Figure S1**. Timeline of serosurveys and vaccination rollout in Geneva, Switzerland

**Figure S2**. Participants recruitment and inclusion into analytical samples

**Figure** **S3**. Reasons for refusal to participate to the study as provided by invited individuals

**Figure** **S4**. Comparison of age and sex composition of study samples (bars) and the Geneva population (dots)

**Figure** **S5**. Antibodies’ response category and vaccination status

**Figure** **S6**. Confirmed SARS-CoV-2 infection cases and estimated seroprevalence of anti-SARS-CoV-2 antibodies in the general population of Geneva, Switzerland, April 2020 to June 2022

**Figure** **S7**. Distribution of ED50% values for all tested SARS-CoV-2 variants by age group

**Figure** **S8**. Seroprevalence of neutralizing antibodies against main SARS-CoV-2 variants in the general population of Geneva, Switzerland, April 29^th^ to June 9^th^, 2022

**Figure** **S9**. Marginal posterior distributions of (A) the probability of any antibody by age group with and without post-stratification to the vaccination levels in the population of Geneva, and (B) the probability of infection by age group and sex

**Figure** **S10**. Marginal posterior distributions for Roche-N and Roche-S sensitivity and specificity

**Figure** **S11**. Marginal posterior distributions of regression coefficients for probability of antibody presence of infection origin and for probability of vaccination

**Figure** **S12**. Neutralization assay results for each (sub)variant versus Roche anti-S values

**Figure** **S13**. Neutralization assay results for each (sub)variant versus Roche anti-S values, stratified by vaccination and infection status

**Table** **S1**. Comparison of education level in the main study sample and in Geneva population

**Table S2.** Comparison of proportion vaccinated in main study sample and in Geneva population

**Table S3.** Prevalence ratio for seroprevalence of anti-SARS-CoV-2 antibodies in Geneva, Switzerland, April 29^th^ to June 9^th^, 2022

**Table S4.** Educational level of sample, serological results, and seroprevalence estimates in Geneva, Switzerland, April 29^th^ to June 9^th^, 2022

**Table S5.** Characteristics of main sample and subsample analyzed in this study

**Table S6.** Demographic characteristics of subsample, serological results, and seroprevalence estimates in Geneva, Switzerland, April 29^th^ to June 9^th^, 2022

**Table S7.** Seroprevalence estimates of neutralizing anti-SARS-CoV-2 antibodies against main variants in the general population of Geneva, Switzerland, April 29^th^ to June 9^th^, 2022

**Table S8.** Comparison of seroprevalence of anti-SARS-CoV-2 antibodies developed through infection by June-July 2021 and April-June 2022, Geneva, Switzerland
